# Nutrient deprivation enhances the cytotoxic effects of a sulfonamide-chalcone hybrid in PC-3 prostate cancer cells

**DOI:** 10.1007/s11010-026-05723-7

**Published:** 2026-09-11

**Authors:** Gisele Santos de Araújo, Rodrigo Elísio de Sá, Stéphanie Aguiar de Negreiros Matos Silva, Fabrício dos Santos Machado, Caridad Noda Perez, Mirian Rita Carrilho de Castro, Anali Del Milagro Bernabe Garnique, Bruna Oliveira de Almeida, Keli Lima, João Agostinho Machado Neto, Letícia Veras Costa Lotufo, José Delano Barreto Marinho Filho, Ana Jérsia Araujo

**Affiliations:** 1 Laboratório de Cultura de Células do Delta, LCCDelta, Universidade Federal do Delta do Parnaíba, UFDPAR, Av. São Sebastião, 2819 - Nossa Senhora de Fátima, Parnaíba, PI 64202-020 Brazil; 2 Instituto de Química, Universidade Federal de Goiás, Goiânia, GO Brazil; 3 Departamento de Farmacologia, Instituto de Ciências Biomédicas, Universidade de São Paulo, São Paulo, SP Brazil

**Keywords:** Androgen-resistant prostate cancer, Nutrient-deprived, Sulfonamide-chalcone hybrid, Metabolic stress

## Abstract

Hybrid molecules integrating different pharmacophoric motifs have emerged as a promising strategy in anticancer drug discovery. In this study, we report for the first time the biological effects of a sulfonamide-chalcone hybrid in the PC-3 prostate cancer cell line under standard culture conditions and nutrient deprivation. The molecular design of SSC185 (µ-(2,5-dichloro-N-{4-[(3E)-4-(3-nitrophenyl)buta-1,3-dien-2-yl]phenyl}benzenesulfonamide) incorporates an α,β-unsaturated system conjugated to the chalcone scaffold together with a sulfonamide moiety, structural features frequently associated with modulation of cancer-related signaling pathways. Results demonstrated that SSC185 reduced the MTT signal of PC-3 cells in a concentration- and time-dependent manner, with IC₅₀ values of 24.69 µM at 24 h and 18.9 µM at 72 h, and exhibited enhanced activity under serum-deprived conditions, with an IC₅₀ of 4.20 µM. Complementary studies employing pharmacological modulators of autophagy, apoptosis, redox status, and proteostasis were conducted to further characterize the compound’s activity. SSC185 increased SQSTM1/p62 and LC3B-I levels without detectable changes in LC3B-II levels or increased acidic vesicle formation, accompanied by reduced AKT/RPS6 signaling and mitochondrial dysfunction. These molecular alterations were associated with the cellular response to SSC185, while nutrient deprivation markedly enhanced its activity in PC-3 cells, indicating that the cellular nutritional context influences the response to the compound.

**Graphical abstract:**

**Figure:**
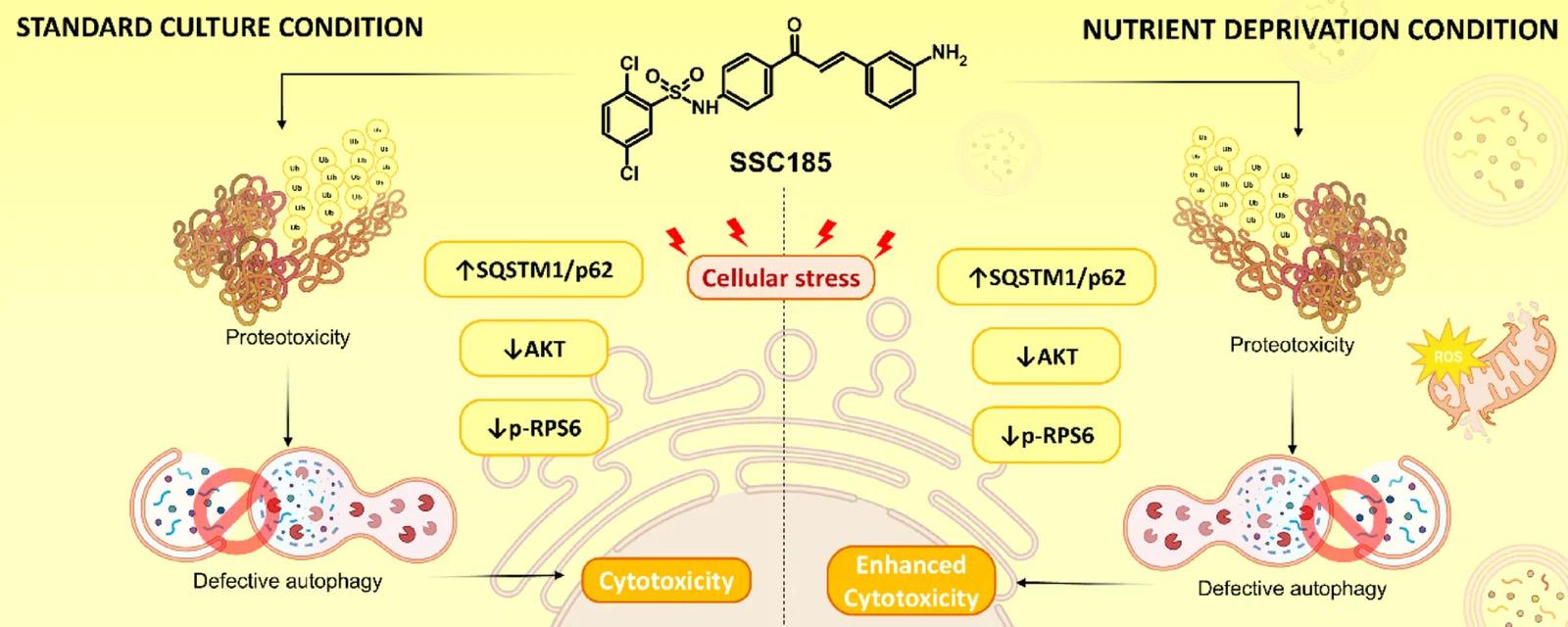

## Introduction

 Identifying relationships between the structural characteristics of small molecules and their biological effects is a cornerstone of modern medicinal chemistry [1]. Therefore, understanding chemical and physicochemical properties influences the interaction of compounds with cellular targets, guiding the development of molecules with greater efficacy and biological selectivity [2]. Among the promising approaches, molecular hybridization has proven to be an established medicinal chemistry strategy that aims to combine two molecules into a new and unique chemical entity, capable of acting through different modes of action and targeting multiple targets simultaneously, resulting in greater therapeutic efficacy and a lower propensity for resistance [3, 4]. In this context, sulfonamide-chalcone hybrids have emerged as attractive scaffolds owing to the combination of complementary pharmacophoric features [5–9]. The α,β-unsaturated system of chalcones can act as an electrophilic center capable of interacting with nucleophilic residues in proteins [10–12], whereas the sulfonamide moiety contributes physicochemical and interaction properties favorable to molecular recognition by biological targets [13–15].

In cancer cells, adaptation to unfavorable conditions involves coordinated regulation of growth signaling, proteostasis, and cellular stress responses, which contribute to survival under conditions such as limited nutrient availability and hypoxia [16–18]. These processes are particularly relevant in advanced prostate cancer, in which tumor cells can maintain proliferation and survival despite adverse environmental conditions [19, 20]. Among the signaling networks involved in these responses, the PI3K/AKT/mTOR pathway integrates growth and nutrient-derived signals and regulates downstream effectors, including RPS6, involved in protein synthesis and cellular growth [21–23]. Beyond growth-related signaling, cellular adaptation to stress also requires mechanisms that maintain protein homeostasis through coordinated protein synthesis, turnover, and degradation [24, 25]. Accordingly, cellular proteostasis involves stress-response mechanisms, including autophagy-related processes in which LC3 and the multifunctional adaptor SQSTM1/p62 participate [26, 27]. Changes in these processes may also involve vesicular compartments, providing complementary information on cellular responses to stress [28]. In addition, nutrient availability can influence redox balance and mitochondrial function, thereby affecting tumor cell responses to pharmacological treatment [29, 30]. Thus, evaluating SSC185 under standard and nutrient-deprived conditions may help determine how the nutritional context influences its cellular effects.

**Fig. 1:**
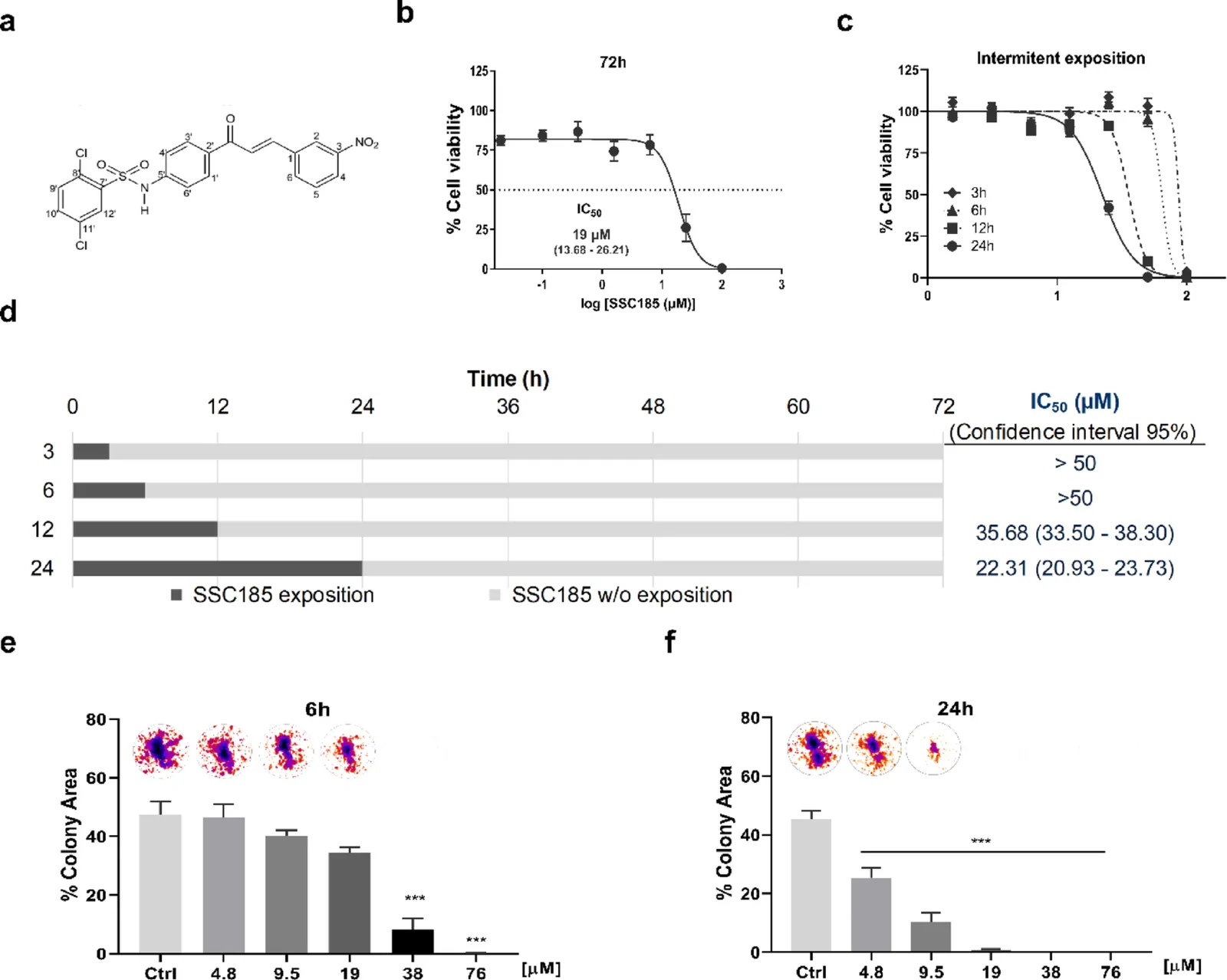
SSC185 reduces the MTT signal and impairs the clonogenic potential of PC-3 cells. **a** Chemical structure of SSC185. **b** Cells were incubated with serial concentrations of SSC185 (up to 100 µM) for 72 h, and the MTT signal was determined. **c** Dose-response curve after intermittent exposure (3, 6, 12, and 24 h) to SSC185, evaluated by the MTT assay. **d** The bar graph shows the exposure time to SSC185 (dark gray bar) and the incubation time in compound-free medium (light gray bar), and the IC50 values are also shown. (**e** and **f**) Graph of the percentage of colony area of PC-3 cells after 6 h and 24 h of exposure to SSC185 at different concentrations (4.8, 9.5, 19, 38, and 76 µM). Data were compared using analysis of variance (ANOVA) followed by Tukey’s post-hoc test. The data are representative of three independent experiments. Bars, S.E.M. ***p < 0.001

SSC185 (µ-(2,5-dichloro-N-{4-[(3E)−4-(3-nitrophenyl)buta-1,3-dien-2-yl]phenyl}benzenesulfonamide) is a sulfonamide-chalcone hybrid synthesized via Claisen-Schmidt condensation and contains an α,β-unsaturated system with potential electrophilic reactivity (Fig. 1a). Previous studies have demonstrated biological activity of SSC185 in different tumor models, including alterations in cell-cycle progression and concentration-dependent apoptosis or necroptosis in SW-620 metastatic lymph node-derived colorectal cancer cells [31], as well as inhibiting adhesion and migration in B16-F10 murine melanoma metastatic cells [32]. These findings prompted further investigation of the cellular effects of SSC185 in other aggressive cancer models. PC-3 cells represent an androgen-independent prostate cancer model characterized by aggressive behavior and alterations in survival signaling pathways, including the PI3K/AKT/mTOR axis, providing a suitable system for further characterization of the cellular responses associated with SSC185.

Initial morphological observations of SSC185-treated PC-3 cells revealed cytoplasmic vacuolization, which prompted further investigation of acidic vesicular compartments and autophagy-related proteins, including LC3B and SQSTM1/p62. Based on these observations and on the cellular processes involved in stress adaptation described above, the present study characterized the effects of SSC185 in PC-3 cells under standard culture conditions and nutrient deprivation. Complementary analyses included cell-cycle distribution, clonogenic capacity, cellular integrity, autophagy-related markers and acidic vesicular compartments, AKT/RPS6 signaling, mitochondrial membrane potential, and responses to pharmacological modulators. This approach allowed the cellular effects of SSC185 to be examined across processes related to proliferative signaling, proteostasis, vesicular organization, mitochondrial function, and nutrient availability.

## Materials and methods

### Reagents and chemicals

SSC185 was synthesized by Claisen-Schmidt condensation at the Chemical Institute, Federal University of Goiás (UFG), Goiânia, Brazil. Its structure was elucidated through high-resolution mass spectroscopy and nuclear magnetic resonance (^1^H and ^13^C NMR), as previously reported by [31, 32]. Dimethyl sulfoxide (DMSO) and 3-(4,5-dimethylthiazol-2-yl)−2,5-diphenyltetrazolium bromide (MTT) were obtained from Exodo científica (Sumaré, SP, Brazil). The Panoptic staining kit was obtained from Laborclin^®^ (Pinhais, PR, Brazil). Neutral red was obtained from Inlab (São Paulo, SP, Brazil). Primary antibodies against phosphorylated PARP1 (#9542, RRID: AB_2160739), phospho-AKT (#4060, RRID: AB_2315049), AKT (#4691, RRID: AB_915783), phospho-RPS6 (#4858, RRID: AB_916156), RPS6 (#2217, RRID: AB_331355), phospho-4E-BP1 (#9455, RRID: AB_560835), 4E-BP1 (#9452, RRID: AB_331692), phospho-ERK1/2 (#9101, RRID: AB_331646), ERK1/2 (#9102, RRID: AB_330744), SQSTM1/p62 (#88588, RRID: AB_2800197), LC3B I/II (#2775, RRID: AB_915950), α-tubulin (#2144, RRID: AB_2210548), and GAPDH (#2118, RRID: AB_561053) were obtained from Cell Signaling Technology (Danvers, MA, USA). Secondary anti-rabbit (#7074, RRID: AB_2099233) and anti-mouse (#7076, RRID: AB_330924) antibodies conjugated with horseradish peroxidase were also obtained from Cell Signaling Technology (Danvers, MA, USA). Chloroquine (CQ), pan-caspase inhibitor Quinoline-Val-Asp-Difluorophenoxymethylketone (Q-VD-OPh), cycloheximide (CHX), N-acetylcysteine (NAC), curcumin, L-cysteine, and DTNB were purchased from Sigma (St. Louis, MO, USA). Propidium iodide and Rhodamine 123 were obtained from Invitrogen (Thermo Fisher Scientific, Waltham, MA, USA).

### Cell lines and culture

The PC-3 cell line (RRID: CVCL_0035), obtained from the American Type Culture Collection (ATCC, CRL-1435), was maintained in RPMI 1640 medium (Sigma-Aldrich, St. Louis, MO, USA) supplemented with 10% fetal bovine serum (FBS; Capricorn Scientific, Ebsdorfergrund, HE, Germany) and 1% penicillin (100 U/mL)-streptomycin (100 µg/mL) (Sigma-Aldrich, St. Louis, MO, USA), in a humidified atmosphere containing 5% CO₂ at 37 °C. The deprivation of FBS induced nutrient deprivation of the PC-3 cell line for 24 h. Cells were seeded overnight, and the supplemented RPMI medium (FBS 10%) was removed. The cells were washed with Phosphate-buffered saline (PBS), and RPMI medium without supplementation was added 24 h before the assays.

### MTT assay

Cell viability was assessed indirectly using the MTT reduction assay [33]. PC-3 cells were seeded in 96-well plates at a density of 5 × 10³ cells/well and allowed to adhere for 24 h. The cells were then treated with increasing concentrations ranging from 0.02 to 100 µM of SSC185 as an initial screening to determine IC_50_ values at 72 h following [34]. After determining the IC_50_ value at 72 h, the 24 h time point was evaluated, with concentrations ranging from 1.6 to 100 µM, to assess the effects of compound exposure over time under standard culture conditions (with FBS) or serum deprivation, in which cells were deprived of FBS for 24 h before treatment. For the inhibition studies, cells were pretreated for 4 h with CQ, Q-VD-OPh, CHX, or NAC. After pretreatment, the inhibitors were removed, and the cells were exposed to the compound for 24 h, both under standard conditions and under serum deprivation conditions. Control groups treated only with inhibitors were included. At the end of exposure to the compound, MTT (0.5 mg/mL) was added, and the cells were incubated for 3 h at 37 °C. Formazan crystals were solubilized with DMSO, and absorbance reading was performed in a microplate reader (Spectramax 190, Molecular devices, CA, USA) at 595 nm.

### Intermittent exposure assay

For intermittent SSC185 exposure, the MTT assay was used. The assay was conducted as described previously [35]. PC-3 cells (5 × 10³ cells/well) were seeded in a 96-well plate. Cells were exposed to SSC185 (1.6 to 100 µM) for 3, 6, 12, or 24 h. After each predetermined time point, the medium containing SSC185 was removed, and the wells were washed with PBS, followed by incubation in compound-free media for 72 h. The MTT solution (0.5 mg/mL) was added to each well and incubated for 3 h. The MTT-formazan product dissolved in DMSO was quantified by measuring absorbance at 595 nm using a microplate reader Spectramax 190 (Molecular devices, CA, USA).

### Colony formation assay

The clonogenic assay was conducted as described previously [34]. 500 cells per well of the PC-3 cell line were seeded in sterile 6-well plates. Concentrations of 4.8, 9.5, 19, 38, and 76 µM of SSC185 were added to the corresponding wells for 6 and 24 h. After the designated time, the cells were washed with PBS, and the medium was promptly replaced with compound-free RPMI for 8 days. The cells were then washed with PBS and stained with a solution of methanol and 1% crystal violet for 5 min. Images were acquired using the G: BOX Chemi XRQ (Syngene, Cambridge, UK) and analyzed using the “ColonyArea” plugin in ImageJ software (National Institutes of Health, Bethesda, MD, USA).

### Cell morphology analysis

Cell morphology was assessed by optical microscopy. PC-3 cells were seeded at a density of 0.5 × 10^5^ cells per well in 24-well plates and allowed to adhere for 24 h. The cells were then treated with SSC185 at concentrations of 19 and 38 µM, using CQ (100 µM) as a control. After 6 h of exposure, the cells were either examined directly by optical microscopy or stained with the Panoptic rapid kit [9] or Neutral Red (40 µg/mL) [36], as previously described, to visualize morphological changes and intracellular vacuolization. Images were acquired using a Zeiss Primovert inverted microscope (Oberkochen, BW, DE) equipped with a 200× total magnification objective.

### Cell cycle distribution and plasma membrane integrity analysis

Cell cycle and plasma membrane integrity analysis was performed by flow cytometry with propidium iodide (PI) DNA labeling following [37]. PC-3 cells were seeded in 12-well plates at a density of 1 × 10⁵ cells per well. Cells were exposed to SSC185 (19 µM and 38 µM) under standard conditions for 6 and 24 h or (4 µM) under starvation conditions for 24 h. The pellets were resuspended in 1 mL of PBS. A 200 µL aliquot of the cell suspension, both exposed and unexposed to the compound, was incubated with 200 µL of lysis solution (0.1% sodium citrate, 0.1% Triton X-100, and 2 µg/mL PI in PBS) for 30 min in the dark to analyze the distribution of DNA content. For the analysis of plasma membrane integrity, PC-3 cells were collected, washed with PBS, and incubated with PI at a final concentration of 25 µg/mL for 10 min at room temperature, protected from light. After incubation, the samples were analyzed immediately. Cells were considered PI-negative when they exhibited preserved plasma membrane integrity. Analyses were performed using a CytoFLEX cytometer (Beckman Coulter, Brea, CA, USA) and the data were analyzed using FlowJo v.X.0.7 software (Treestar, Inc., San Carlos, CA, USA).

### Mitochondrial depolarization analysis

Mitochondrial depolarization analysis was performed by flow cytometry using Rhodamine 123 (Rhd 123), as described previously [38]. Briefly, PC-3 cells were seeded in 12-well plates at a density of 1 × 10⁵ cells per well. Cells were exposed to SSC185 (19 µM and 38 µM) under standard conditions for 6 and 24 h or (4 µM) under starvation conditions for 24 h. The pellets were resuspended in 1 mL of PBS. A 500 µL aliquot of cell suspensions exposed and unexposed to the compound was centrifuged in PBS for staining. Staining was performed with Rhd 123 added to the cell suspension at a final concentration of 1 µM. Samples were incubated for 15 min in the cold, protected from light. After incubation, the cells were washed with cold 1× PBS and centrifuged (2,000 rpm/5 minutes) to remove excess dye and resuspended in 500 µL of PBS for acquisition. The acquisition was performed using a CytoFLEX cytometer (Beckman Coulter, Brea, CA, USA), and the data were analyzed using the FlowJo software v.X.0.7 (Treestar, Inc., San Carlos, CA, USA).

### Acidic vesicular organelles (AVOs) stained by acridine orange

The assessment of acidic vesicular organelles was performed as previously described [39]. PC-3 cells were seeded in 24-well plates at a density of 1 × 10⁵ cells per well. Cells were exposed to SSC185 (19 µM and 38 µM) under standard conditions for 24 h or (4 µM) under nutrient deprivation conditions for 24 h. Cells were washed with PBS and incubated with a solution containing 0.1 µg/mL acridine orange (AO) (Sigma-Aldrich) in PBS for 15 min. After staining, cells were analyzed using GFP and RFP channels on a Lionheart FX automated fluorescence microscope (Agilent BioTek Instruments Inc., Santa Clara, CA, USA) at 200× magnification.

### Western blotting

Protein expression was analyzed by Western blotting, as described previously [40]. Total protein extraction from PC-3 prostate cancer cells was performed using a buffer containing 100 mM Tris (pH 7.6), 1% Triton X-100, 2 mM PMSF, 10 mM Na3VO4, 100 mM NaF, 10 mM Na4P2O7, and 4 mM EDTA, from cells with or without exposure to SSC185 (9, 5, 19, and 38 µM) for 24 h. The extracted proteins were quantified using the Bradford method. After sodium dodecyl sulfate-polyacrylamide gel electrophoresis (SDS-PAGE) (30 µg), the proteins were transferred to nitrocellulose membranes (GE Healthcare, Chicago, IL, USA) and detected by incubating the membranes with specific primary antibodies and horseradish peroxidase-conjugated secondary antibodies. Protein bands were detected using the SuperSignalWest Dura Extended Duration substrate system (Thermo Fisher Scientific, Inc.) and a G: BOX Chemi XX6 gel documentation system (Syngene). Protein bands were normalized relative to tubulin or GAPDH.

### Thiol reactivity assay

The thiol reactivity of SSC185 was evaluated using L-cysteine and the DTNB/Ellman reagent, as previously described [41], with minor modifications. SSC185 or curcumin (25 or 50 µM) was incubated with L-cysteine (250 µM) in 50 mM phosphate buffer, pH 7.4, at room temperature, in 96-well plates, for 0, 5, 10, 15, 30, 60, and 120 min. After each incubation period, DTNB was added to a final concentration of 500 µM, and the mixture was incubated for 5 to 10 min, protected from light. Absorbance was measured at 405 nm using a microplate reader (DR-200BN-BI, Kasuaki, Brazil distributor).

Wells containing only L-cysteine were used as free thiol controls with their respective incubation times, while wells containing phosphate buffer and DTNB were used for background signal correction. Additional wells containing SSC185 or curcumin, in the absence of L-cysteine, were included to correct for the intrinsic absorbance of the compound. Curcumin was used as an electrophilic reference compound. The remaining free L-cysteine was expressed as a percentage relative to the corresponding L-cysteine control, according to the following equation:

$$ \begin{aligned} \% ~ & remaining~free~L - cysteine \\ & = \frac{{\left( {A_{{sample}} - A_{{compound~control}} } \right)}}{{\left( {A_{{L~ - ~cysteine~control~}} - A_{{blank}} } \right)}} \times 100 \\ \end{aligned} $$

### Statistical analysis

The statistical studies were performed using GraphPad Prism (Institutive Software for Science, San Diego, California, USA). MTT signal was expressed as a percentage of untreated controls, and IC₅₀ values were obtained by non-linear regression. The data were compared using analysis of variance (ANOVA) followed by Tukey’s post-hoc test. Data are representative of three independent experiments. Bars, Standard Error Mean (S.E.M.); *p < 0.05, **p < 0.01, ***p < 0.001.

## Results

### SSC185 reduces the MTT signal and clonogenic capacity of PC-3 cells in a time- and concentration-dependent manner

To investigate the biological effects of SSC185 in androgen-independent PC-3 prostate cancer cells, we first evaluated its effects using the MTT reduction assay. SSC185 reduced the MTT signal with an IC₅₀ of 19 µM after 72 h of continuous exposure (Fig. 1b). When PC-3 cells were treated for 24 h, SSC185 reduced the MTT signal in a concentration-dependent manner, with an IC₅₀ of 24.69 µM (Fig. 6a), indicating that the compound exerts a relevant effect in the first 24 h of exposure.

To further characterize the temporal dependence of SSC185 activity, an intermittent exposure assay was performed in which PC-3 cells were exposed to SSC185 for 3, 6, 12, or 24 h. The medium containing the compound was then replaced with compound-free medium until a total incubation time of 72 h was reached. The MTT signal was then quantified. Short exposure intervals of 3 and 6 h required markedly higher concentrations of SSC185 (IC₅₀ > 50 µM) to reduce the MTT signal (Fig. 1c, d). In contrast, exposures of 12 h and 24 h resulted in IC₅₀ values of 35.68 µM and 22.31 µM, respectively (Fig. 1c, d). These results indicate that the effect of SSC185 on MTT reduction depends on both exposure time and concentration, with longer exposure resulting in lower IC₅₀ values.

The long-term proliferative potential of PC-3 cells after transient exposure to SSC185 was evaluated using a clonogenic assay. Cells were treated with SSC185 (4.8, 9.5, 19, 38, and 76 µM) for 6–24 h, after which the medium was replaced with compound-free medium and colonies were allowed to form. The percentage of colony area was then quantified. After 6 h of treatment, a significant reduction in colony formation was observed only at 38 and 76 µM (*p* < 0.001), whereas no significant reduction was detected at 4.8, 9.5, or 19 µM (Fig. 1e).

In contrast, after 24 h of exposure, a significant reduction in colony formation was observed across a broader concentration range. Even the lowest concentration tested (4.8 µM) significantly reduced colony formation (*p* < 0.001), while complete suppression of colony formation was observed at concentrations ≥ 19 µM (*p* < 0.001; Fig. 1f).

### SSC185 promotes morphological changes and reduces plasma membrane integrity

**Fig. 2:**
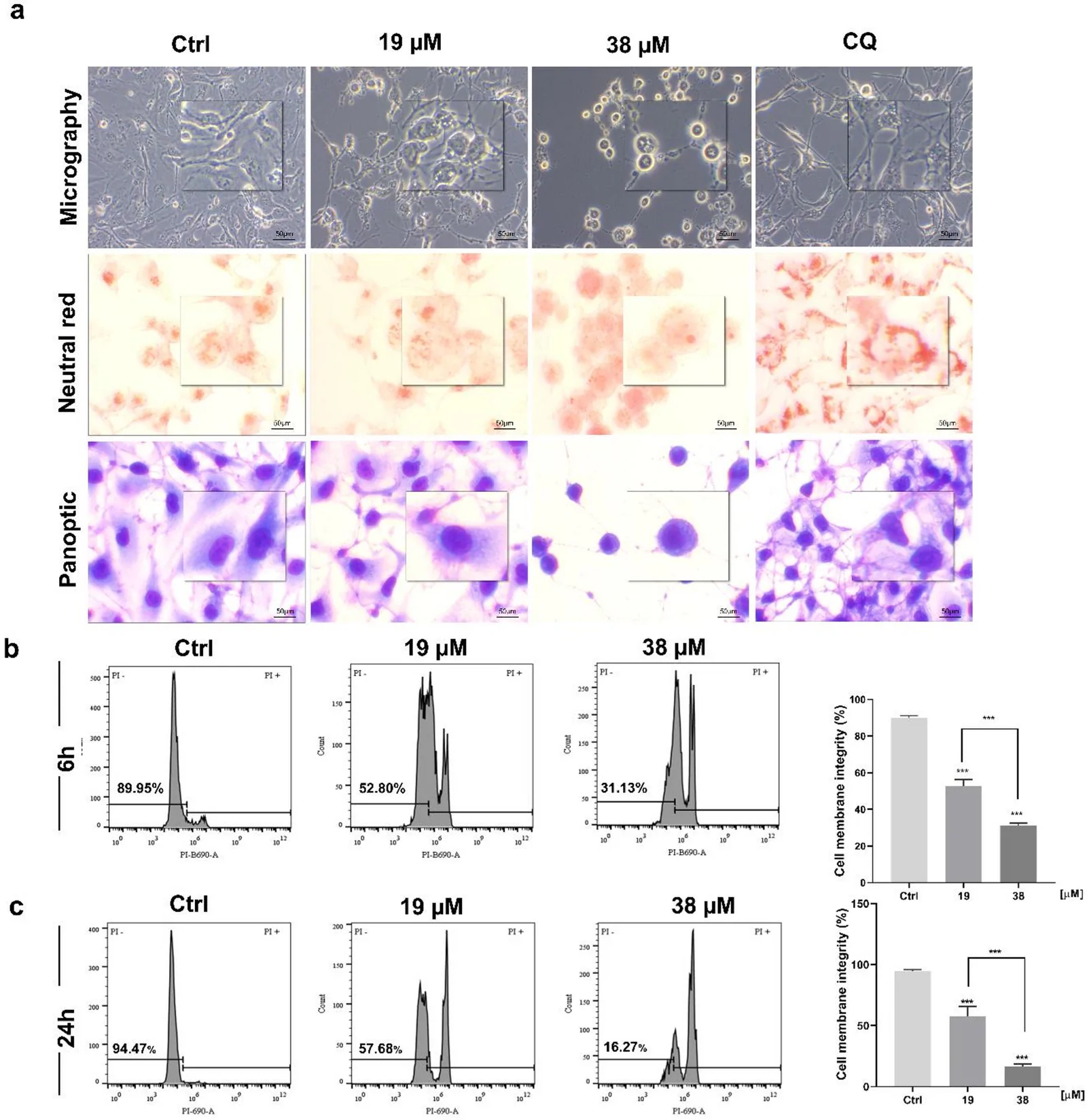
SSC185 induces changes in the morphology of PC-3 cells with vacuole formation and loss of membrane integrity. **a** Representative images of the cell morphology assay of the PC-3 cell line after exposure to SSC185 at concentrations of 19 and 38 µM for 6 h. Chloroquine (100 µM) was used as a positive control, inducing cytoplasmic vacuolization. Images were enlarged to highlight cytoplasmic vacuolization. Escale (50 μm). **b** Representative histograms of cells stained with PI and a bar graph demonstrating the percentage of cells with intact membranes after exposure to SSC185 at concentrations of 19 and 38 µM for 6 h. **c** Representative histograms of cells stained with PI and a bar graph demonstrating the percentage of cells with intact membranes after exposure to SSC185 at concentrations of 19 and 38 µM for 24 h. Data were compared using analysis of variance (ANOVA) followed by Tukey’s post-hoc test. The data are representative of three independent experiments. Bars, S.E.M; **p < 0.01, ***p < 0.001. Ctrl: Negative control; CQ: Chloroquine; PI: Propidium iodide

Next, the initial effects of SSC185 on the morphology and plasma membrane integrity of PC-3 cells were evaluated during the first 6 h of exposure. Plasma membrane integrity was assessed based on the proportion of PI− cells, which correspond to cells retaining the ability to exclude propidium iodide. Neutral red and rapid panoptic staining revealed concentration-dependent morphological changes. Treatment with SSC185 at 19 µM (IC₅₀) induced cytoplasmic vacuolization (Fig. 2a) and reduced the proportion of PI-negative (PI−) cells to 52.80% (*p* < 0.001; Fig. 2b), compared with 89.95% in the control group. At 38 µM (2× IC₅₀), cells exhibited retraction and prominent cytoplasmic vacuoles with intense basophilia (Fig. 2a), accompanied by a further reduction in the PI− population to 31.13% (*p* < 0.001; Fig. 2b). These findings indicate a concentration-dependent reduction in the proportion of cells retaining plasma membrane integrity. Although cytoplasmic vacuolization was observed at both 19 and 38 µM, the neutral red staining pattern differed from that observed following chloroquine treatment (CQ, 100 µM), indicating distinct patterns of vacuolar staining under these experimental conditions (Fig. 2a).

After 24 h of treatment, the proportion of PI− cells was reduced to 57.68% following exposure to 19 µM SSC185 (*p* < 0.001) and to 16.27% following exposure to 38 µM SSC185 (*p* < 0.001), compared with 94.47% in the control group (Fig. 2c). The marked reduction in the PI− population at 38 µM indicates a pronounced loss of plasma membrane integrity after prolonged exposure.

### SSC185 induces time‑ and concentration‑dependent cell cycle alterations

**Fig. 3:**
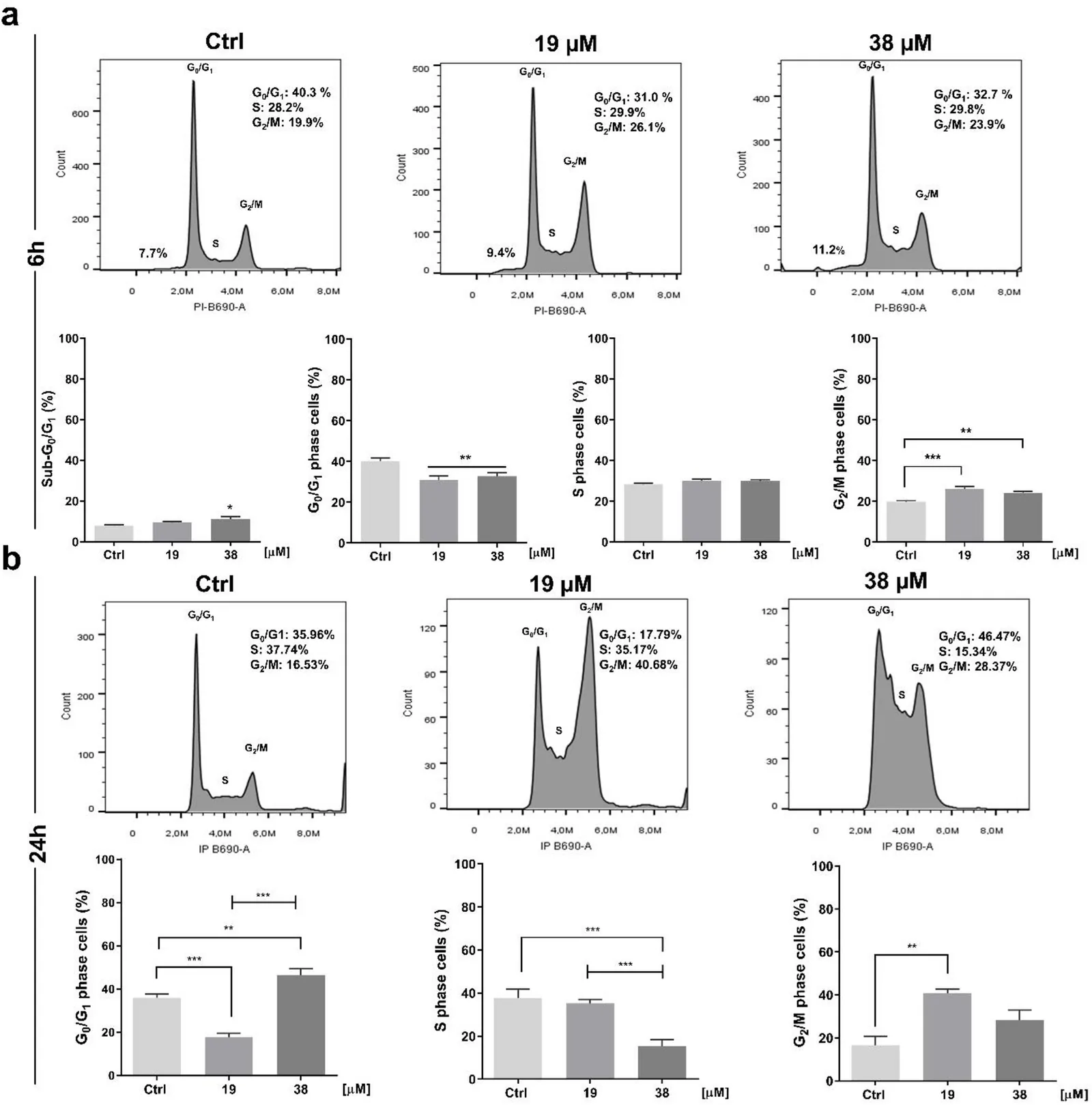
SSC185 induces time- and concentration-dependent alterations in cell cycle distribution in PC-3 cells. **a** Representative histograms of DNA content distribution and a graph of the percentage of PC-3 cells in different cell cycle phases after exposure to SSC185 at concentrations of 19 and 38 µM for 6 h. **b** Representative histograms of DNA content distribution and a graph of the percentage of PC-3 cells in different cell cycle phases after exposure to SSC185 at concentrations of 19 and 38 µM for 24 h. Data were compared by analysis of variance (ANOVA) followed by Tukey’s post-hoc test. Data are representative of three independent experiments. Bars, S.E.M.; *p < 0.05, **p < 0.01, ***p < 0.001

Given that SSC185 impaired viability, morphology, and membrane integrity, its impact on cell cycle progression was evaluated in PC‑3 cells. Flow cytometry analysis demonstrated time- and concentration-dependent alterations in cell cycle distribution. After 6 h of treatment, SSC185 reduced the G_0_/G_1_ fraction and increased the proportion of cells with 4 C DNA content, consistent with accumulation in the G_2_/M phase. The proportion of cells in G_2_/M increased to 26.13% ± 1.00% at 19 µM and 23.93% ± 0.94% at 38 µM (*p* < 0.001), compared with 19.85% ± 0.39% in control cells (Fig. 3a). Correspondingly, the G_0_/G_1_ population decreased from 40.27% ± 1.31% in control cells to 31.04% ± 1.78% and 32.70% ± 1.72% at 19 and 38 µM, respectively (*p* < 0.01; Fig. 3a).

After 24 h, the effects diverged according to concentration. At 19 µM, the proportion of cells in G_2_/M remained elevated and further increased to 40.68% ± 2.01% (*p* ≤ 0.01; Fig. 3b). In contrast, at 38 µM, cells shifted predominantly to G_0_/G_1_ (46.47% ± 3.01%, *p* ≤ 0.01), accompanied by a reduction in the S‑phase fraction (17.79% ± 1.64%, *p* ≤ 0.01; Fig. 3b). Notably, the sub‑G_0_/G_1_ population already increased within the first 6 h at 38 µM, from 7.76% ± 0.50% in control cells to 11.21% ± 1.08% (*p* < 0.05), suggesting early onset of cell death-associated DNA fragmentation at the higher concentration.

Together, these data indicate that prolonged exposure to 19 µM SSC185 is associated with a sustained increase in the proportion of cells in G_2_/M, whereas treatment with 38 µM produces a distinct cell-cycle distribution characterized by increased G_0_/G_1_ and reduced S-phase fractions.

### Exposure to SSC185 does not induce accumulation of acridine orange-stained acidic vesicles

**Fig. 4:**
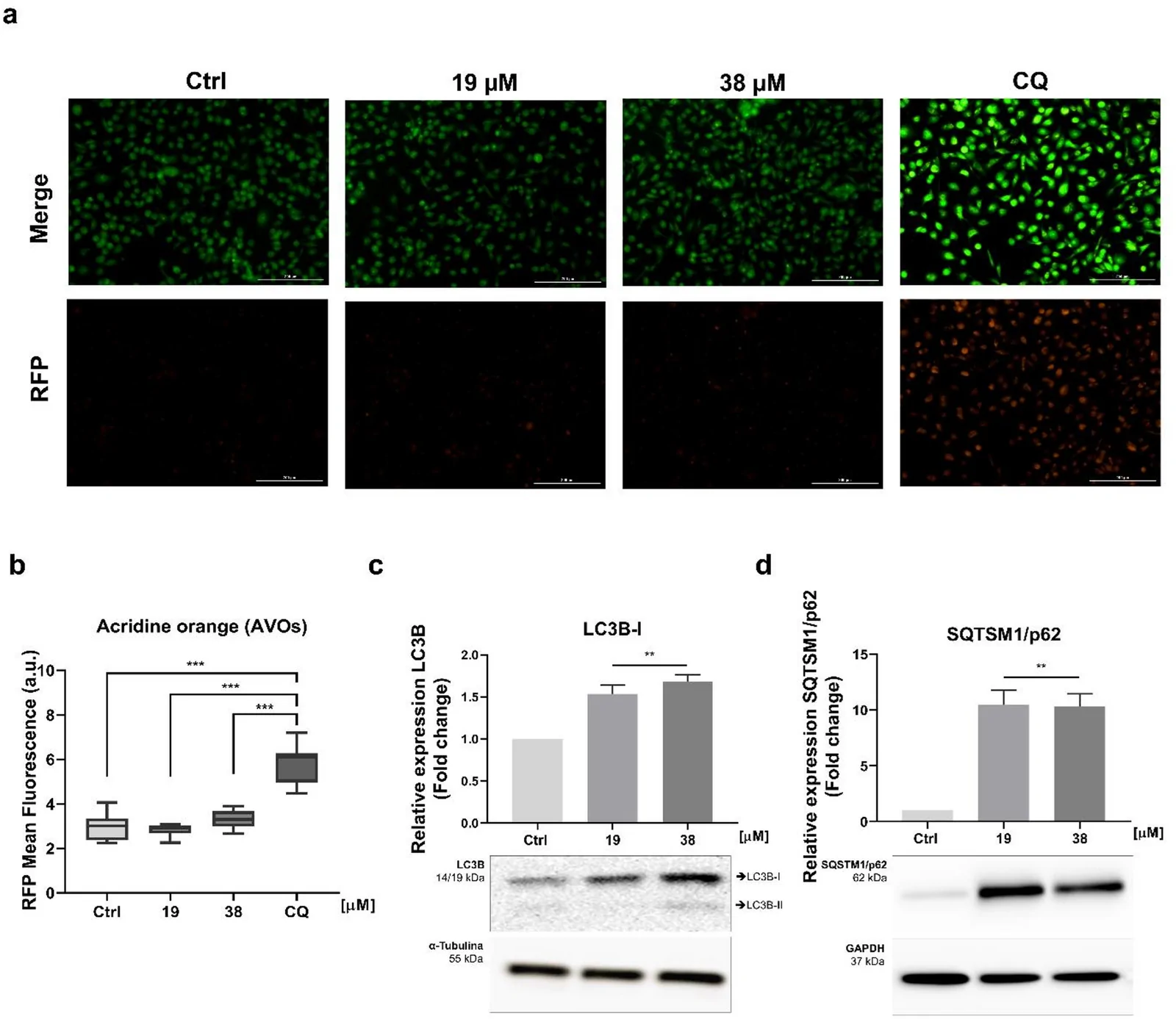
SSC185 induced accumulation of SQSTM1/p62 without significant acidic vesicle formation. **a** Representative images of PC-3 cells stained with AO to assess the presence of acidic vesicular organelles. PC-3 cells were treated with SSC185 at concentrations of 19 µM and 38 µM for 24 h. CQ (100 µM) was used as a control. **b** The graph shows the average fluorescence in the RFP channel of the assay with AO. **c** Representative blots and analysis of the expression levels of LC3BI/II in extracts of PC-3 cells exposed to SSC185 at concentrations of 19 and 38 µM for 24 h. **d** Representative blots and analysis of the expression levels of SQSTM1/p62 in extracts of PC-3 cells exposed to SSC185 at concentrations of 19 and 38 µM for 24 h. Membranes were incubated with antibodies for the detection of target proteins and the loading controls GAPDH and/or tubulin. Data were compared using analysis of variance (ANOVA) followed by Tukey’s post-hoc test. The data are representative of three independent experiments. Bars, S.E.M; *p < 0.05, **p < 0.01, ***p < 0.001. AO: Acridine orange

Based on the cytoplasmic vacuolization observed after SSC185 treatment, acidic vesicular organelles were evaluated by acridine orange fluorescence microscopy. PC-3 cells were exposed to SSC185 at 19 and 38 µM for 24 h, while chloroquine (100 µM) was included as a reference condition (Fig. 4a). Quantitative analysis of the mean fluorescence intensity in the RFP channel showed no significant increase in SSC185-treated cells compared with the untreated control (*p* > 0.05; Fig. 4b). In contrast, chloroquine treatment produced a significant increase in RFP fluorescence (*p* < 0.01), indicating a greater accumulation of acidic vesicular compartments under this condition.

The expression of the autophagy-related proteins LC3B and SQSTM1/p62 was subsequently evaluated by Western blotting. SSC185 treatment significantly increased LC3B-I levels (*p* < 0.01; Fig. 4c), whereas no detectable alteration in LC3B-II levels was observed. Concurrently, SQSTM1/p62 expression was significantly increased following SSC185 exposure (*p* < 0.01; Fig. 4d). Collectively, these results demonstrate that SSC185 induces changes in autophagy-related markers characterized by increased LC3B-I and SQSTM1/p62 levels, without detectable LC3B-II accumulation or an increase in acridine orange-stained acidic vesicles under the experimental conditions evaluated.

### SSC185 modulates pathways involved in cellular survival and metabolism

**Fig. 5:**
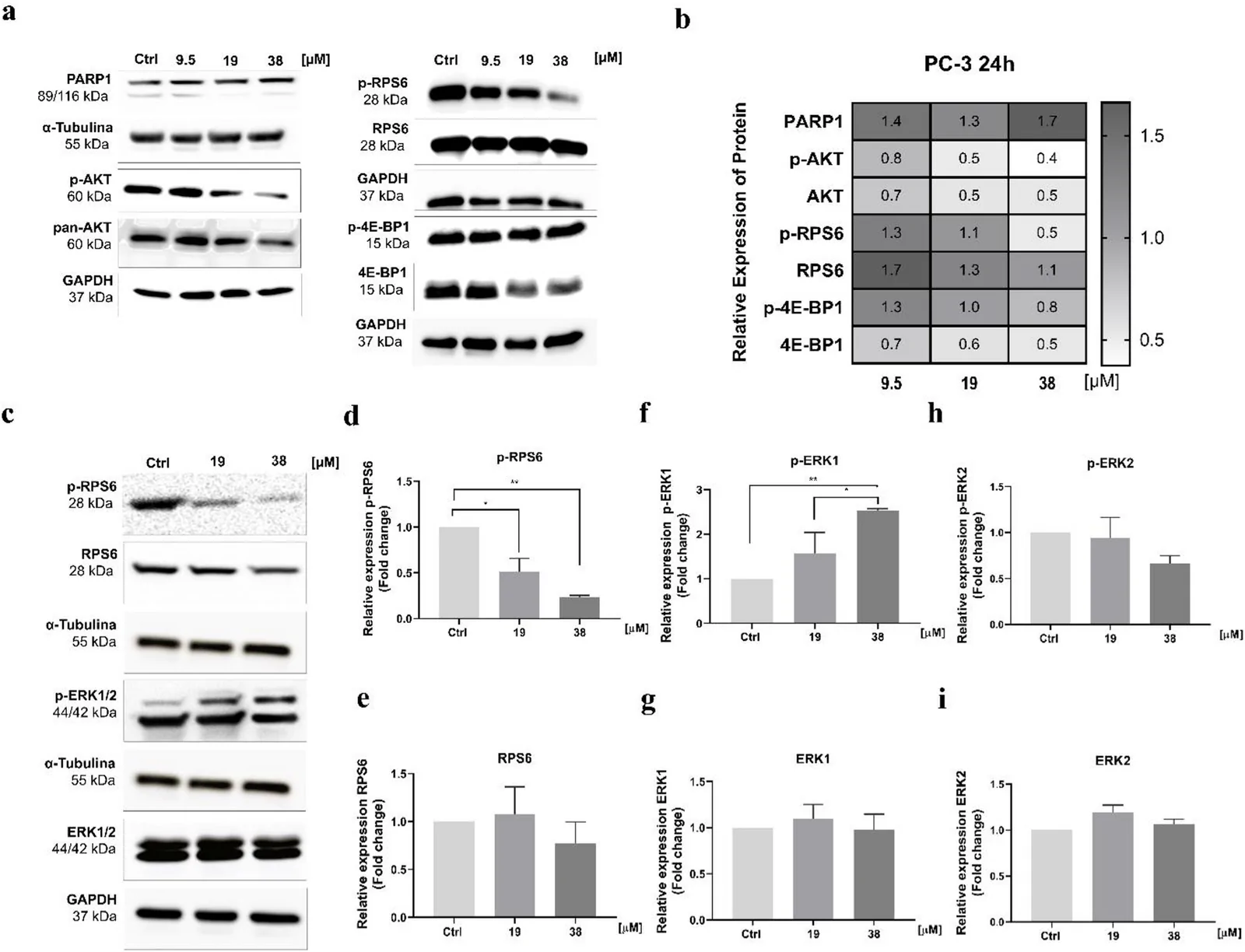
SSC185 alters AKT/RPS6-related signaling and increases p-ERK1 levels in PC-3. **a** Exploratory Western blot analysis of PARP1, p-AKT, pan-AKT, p-RPS6, RPS6, p-4E-BP1, and 4E-BP1 in total extracts of PC-3 cells treated with SSC185 (9.5, 19, and 38 µM) for 24 h. GAPDH and α-tubulin were used as loading controls. **b** Bands were quantified by densitometry and normalized to loading controls; the values presented refer to this single exploratory experiment and are not based on replicate experiments or statistical analyses. **c** Representative blots of the expression levels of phospho-RPS6, RPS6, phospho-ERK1/2, and ERK1/2 in extracts of PC-3 cells exposed to SSC185 at concentrations of 19 and 38 µM for 24 h. **d** Western blot analysis of the expression levels of phospho-RPS6, **e** RPS6, **f** phospho-ERK1, **g** ERK1, **h** phospho-ERK2, and **i** ERK2 in extracts of PC-3 cells exposed to SSC185 at concentrations of 19 and 38 µM for 24 h. Membranes were incubated with antibodies for the detection of target proteins and the loading controls GAPDH and/or tubulin. Data were compared using analysis of variance (ANOVA) followed by Tukey’s post-hoc test. The data are representative of three independent experiments. Bars, S.E.M; *p < 0.05, **p < 0.01, ***p < 0.001

To gain an initial understanding of the mechanisms underlying SSC185-induced cytotoxicity, an exploratory screening of proteins associated with key cell death and survival pathways was performed. PC-3 cells were treated with SSC185 (9.5, 19, and 38 µM) for 24 h, and total protein extracts were analyzed by Western blot. Membranes were probed for PARP1, phosphorylated AKT (p-AKT), total AKT (pan-AKT), phosphorylated RPS6 (p-RPS6), total RPS6, phosphorylated 4E-BP1 (p-4E-BP1), and total 4E-BP1, using GAPDH and α-tubulin as loading controls. Representative blot images are shown in Fig. 5a, and densitometric quantifications are presented in Fig. 5b. In this exploratory analysis, no PARP1 cleavage was detected after 24 h of treatment, while decreases in p-AKT, pan-AKT, p-RPS6, and 4E-BP1 levels were observed. As these initial observations were obtained from a single biological experiment, they were considered exploratory and were not subjected to statistical analysis.

Based on these initial observations, RPS6 signaling was subsequently evaluated in three independent experiments, together with ERK1/2 (Fig. 5c) signaling, considering the central role of the PI3K/AKT/mTOR and MAPK/ERK pathways in maintaining cell survival and proliferative capacity. Western blot analysis (Fig. 5d) showed that exposure to SSC185 significantly reduced the levels of phosphorylated ribosomal protein S6 (p-RPS6) (*p* < 0.01), a downstream effector of the mTOR pathway, compared to the control group. Total S6 protein expression levels were not affected (Fig. 5e). In contrast, under the same treatment conditions, a significant increase in phosphorylated ERK1 (p-ERK1) was observed (Fig. 5f; *p* < 0.01), while the levels of phosphorylated ERK2 (p-ERK2) and total ERK1/2 remained unchanged (Fig. 5g, h, i).

### Nutrient deprivation enhances the cellular response to SSC185 and increases its sensitivity to NAC pretreatment

Nutrient deprivation was used to assess whether the cellular response to SSC185 was influenced by nutritional conditions. Additionally, pharmacological modulators targeting processes related to autophagy (CQ), caspase activity (Q‑VD‑OPh), protein synthesis (CHX), and redox-sensitive (NAC) responses were employed to further characterize the response profile to SSC185 under standard and nutrient-deprived conditions.

**Fig. 6:**
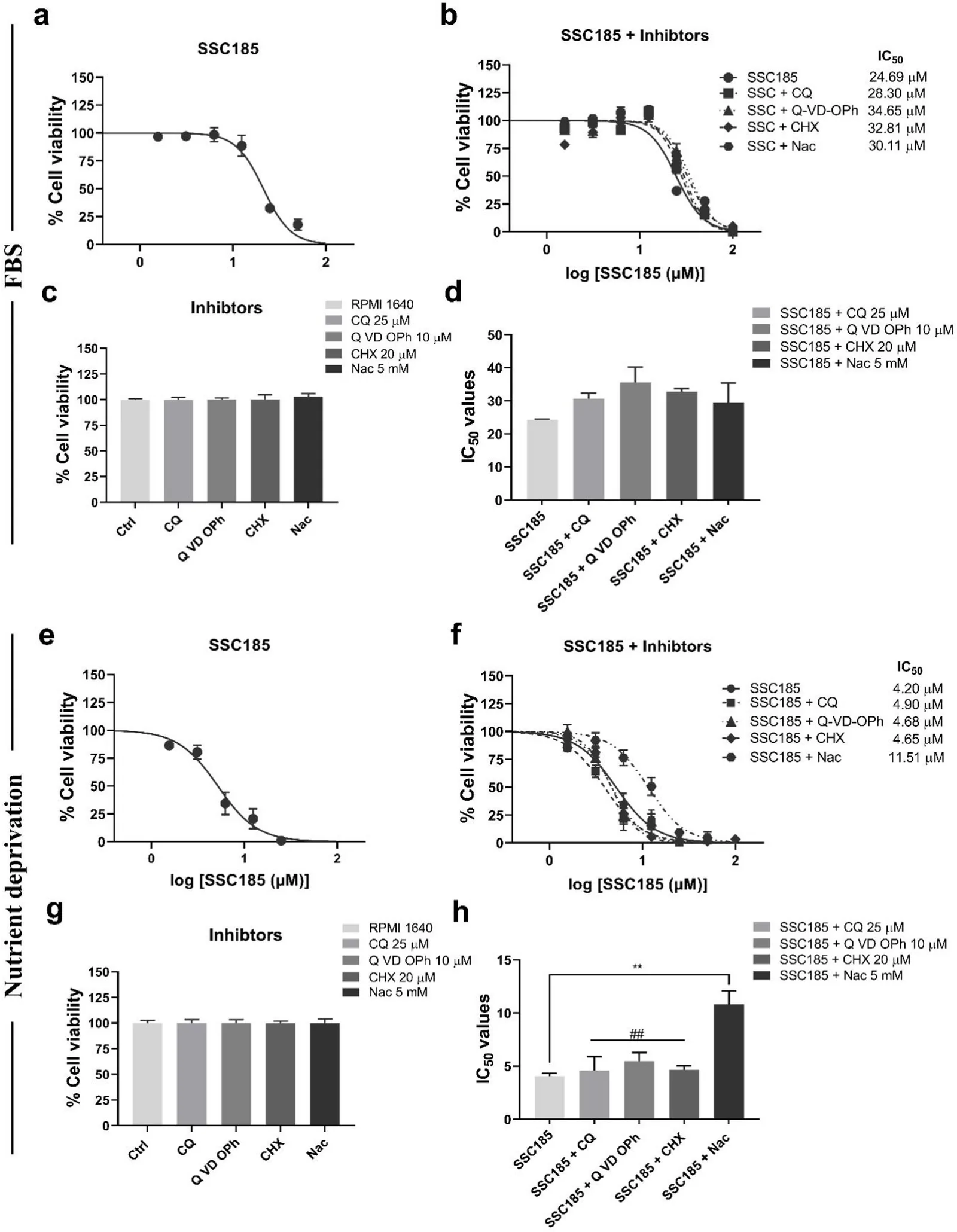
Prostate cancer cells respond differently to SSC185 under standard and nutrient-deprived conditions. **a** MTT dose-response curve obtained after 24 h of treatment with SSC185 at different concentrations, ranging from 1.6 to 100 µM. **b** MTT dose-response curves of SSC185 associated with 4 h pretreatment with CQ, QVD, CHX, and NAC. **c** MTT signal of cells exposed to the inhibitors alone was used as an intrinsic toxicity control. **d** Comparison of SSC185 IC₅₀ values after pretreatment with pharmacological inhibitors. Graphs **a** – **d** correspond to standard culture conditions. **e** MTT dose-response curve obtained after 24 h of treatment with SSC185 at different concentrations, ranging from 1.6 to 100 µM. **f** MTT dose-response curves of SSC185 associated with 4 h pretreatment with CQ, QVD, CHX, and NAC. **g** MTT signal of cells exposed to the inhibitors alone was used as an intrinsic toxicity control. **h** Comparison of IC₅₀ values of SSC185 cells after pretreatment with pharmacological inhibitors. Plots **e** – **h** correspond to nutrient deprivation conditions. Data were compared using analysis of variance (ANOVA) followed by Tukey’s post-hoc test. Data are representative of three independent experiments. Bars, S.E.M; *p < 0.05, **p < 0.01, ***p < 0.001. ## *p* < 0.001 compared to NAC. CQ: chloroquine; CHX: Cycloheximide; NAC: N-acetylcysteine; QVD: Q-VD-OPh

Under standard culture conditions supplemented with FBS, pretreatment with these pharmacological inhibitors did not produce significant changes in IC₅₀ values compared with SSC185 alone (Fig. 6b, d). In contrast, when the assay was performed under starvation conditions, a marked reduction in IC₅₀ was observed, from 24.69 µM (Fig. 6a) to 4.20 µM (Fig. 6e), indicating that nutrient deprivation substantially enhances the effect of SSC185 on MTT reduction. Notably, under starvation, pretreatment with NAC partially attenuated this effect, increasing the IC₅₀ to 11.51 µM (*p* < 0.01) (Fig. 6f, h).

### SSC185 reduces mitochondrial membrane potential under standard and nutrient-deprived conditions

**Fig. 7:**
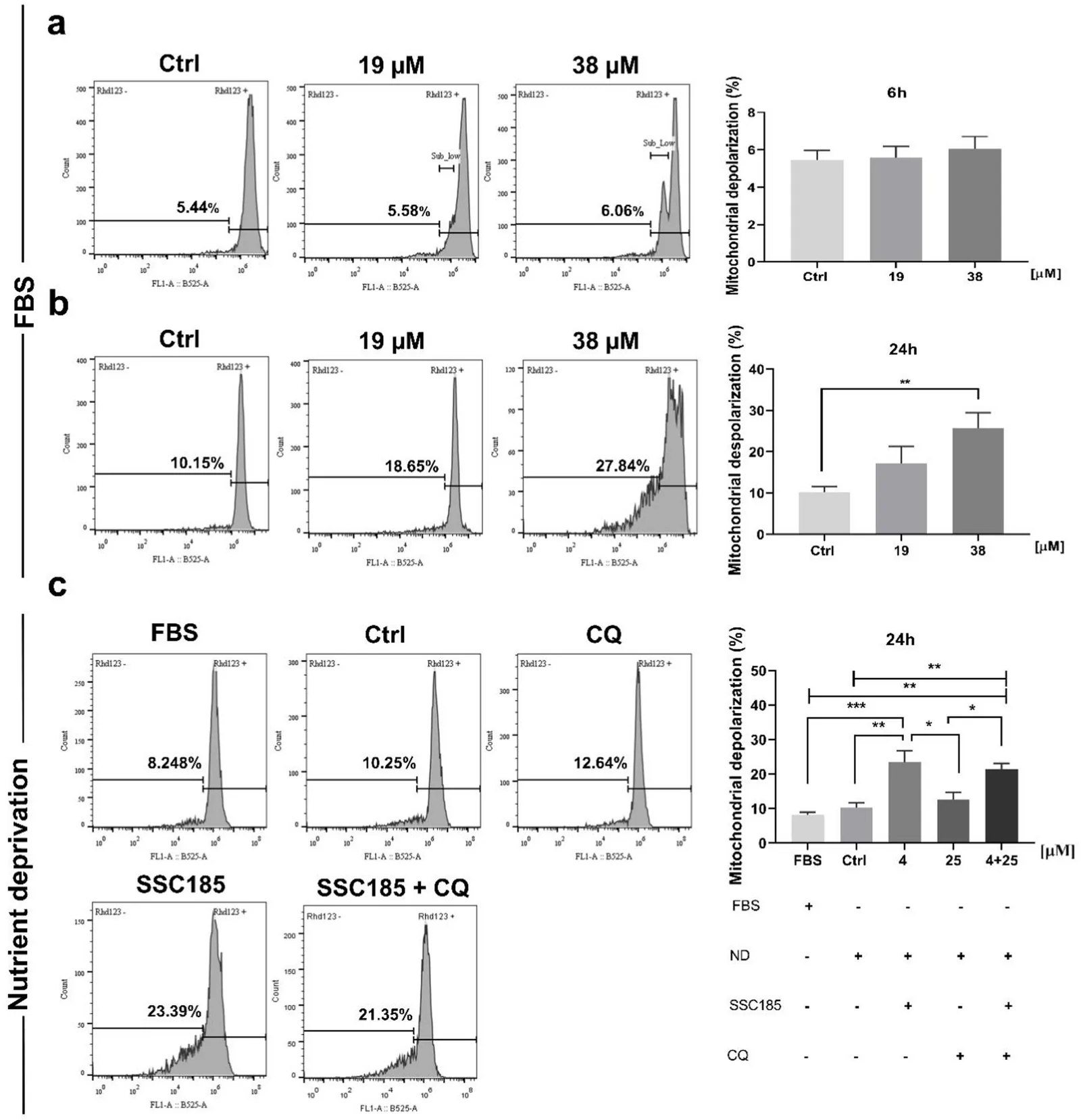
SSC185 induces concentration- and time-induced mitochondrial depolarization, accentuated under nutrient deprivation conditions. **a** Representative histograms of mitochondrial depolarization of PC-3 cells stained with Rhd 123 and a graph showing the percentages of cells with depolarized mitochondrial membranes after exposure to SSC185 at concentrations of 19 and 38 µM for 6 h. **b** Representative histograms of mitochondrial depolarization of PC-3 cells stained with Rhd 123 and a graph showing the percentages of cells with depolarized mitochondrial membranes after exposure to SSC185 at concentrations of 19 and 38 µM for 24 h. A and B represent standard culture conditions. **c** Representative histograms of mitochondrial depolarization of PC-3 cells stained with Rhd 123 and a graph showing the percentages of cells with depolarized mitochondrial membranes after exposure to SSC185 at a concentration of 4 µM, 25 µM of CQ, and co-treated with SSC185 and CQ (4 µM + 25 µM) for 24 h under nutrient deprivation conditions. Data were compared by analysis of variance (ANOVA) followed by Tukey’s post-hoc test. Data are representative of three independent experiments. Bars, S.E.M.; *p < 0.05, **p < 0.01, ***p < 0.001. CQ: chloroquine. ND: Nutrient deprivation

To evaluate mitochondrial responses to SSC185, mitochondrial membrane potential (ΔΨm) was assessed using rhodamine 123. After 6 h of exposure, no statistically significant differences in the proportion of Rho123 − cells were observed between SSC185-treated groups and the control group (Fig. 7a). After 24 h of exposure, treatment with 38 µM SSC185 significantly increased the proportion of Rho123 − cells to 27.84% ± 3.52 (*p* < 0.01), indicating a reduction in mitochondrial membrane potential (Fig. 7b).

Under nutrient deprivation, treatment with 4 µM SSC185 also significantly increased the Rho123 − population to 23.39% ± 3.44 (*p* < 0.01), compared with cells maintained in complete medium (8.24% ± 0.74) and nutrient-deprived controls (10.25% ± 1.44). Co-treatment with chloroquine (CQ, 25 µM) did not prevent the increase in the Rho123 − population associated with SSC185 treatment (Fig. 7c), whereas CQ alone did not significantly alter the proportion of Rho123 − cells compared with the corresponding controls.

### Nutrient deprivation dissociates the effects of SSC185 on the cell cycle of PC-3

**Fig. 8:**
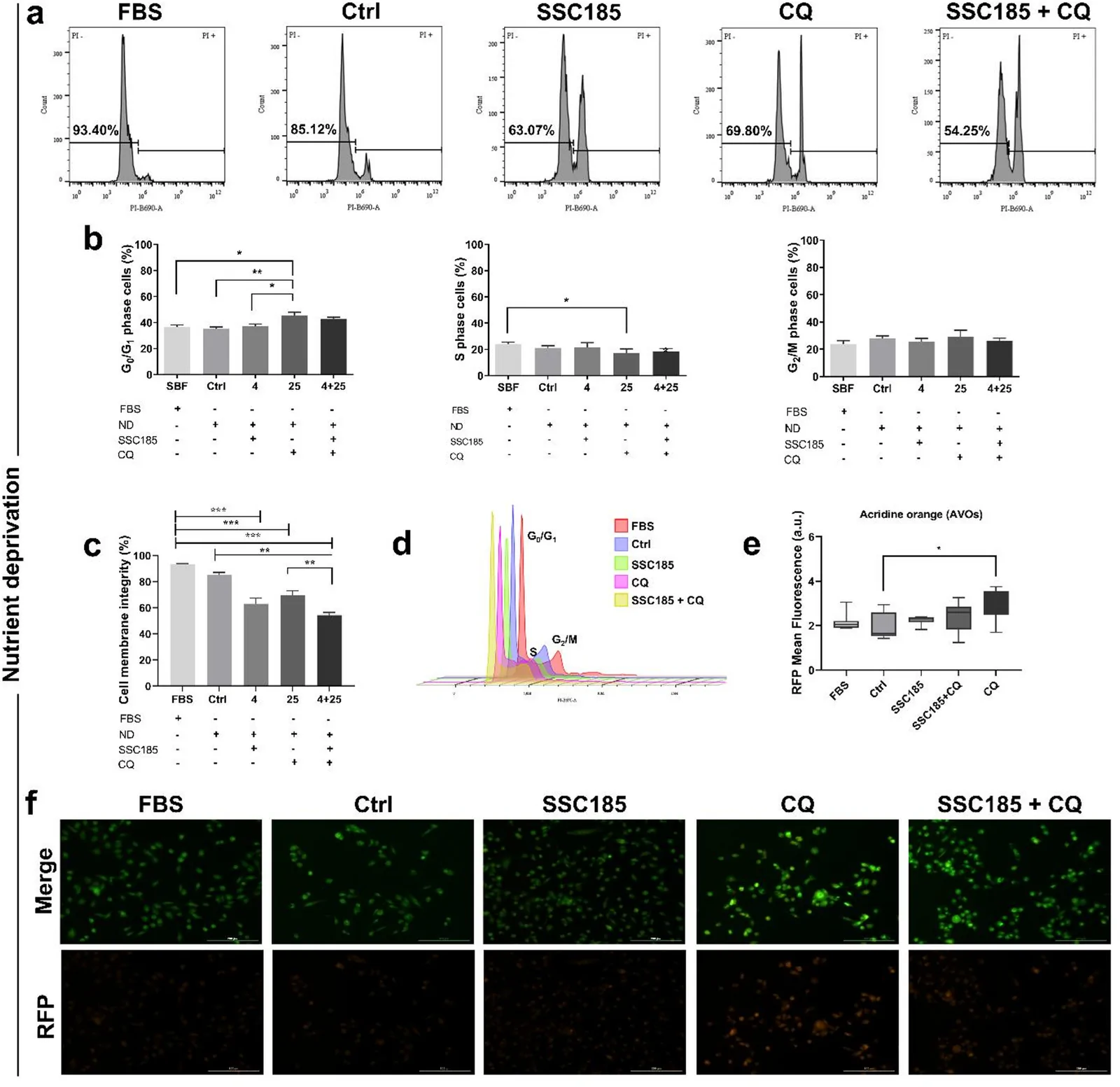
SSC185 compromises membrane integrity without altering the distribution of cell cycle phases in nutrient deprivation-treated prostate cancer cells. **a** Representative histograms of propidium iodide-stained cells, showing cells with intact membranes after exposure to SSC185 (4 µM), CQ (25 µM), and SSC185 + CQ (4 µM + 25 µM) after 24 h. **b** Bar graph demonstrating the percentage of cells with intact membranes after exposure to SSC185. **c** Histogram of the distribution of DNA content in different groups of PC-3 cells after 24 h of treatment. **d** Graph of the percentage of PC-3 cells in different cell cycle phases, exposed to SSC185 at a concentration of 4 µM, 25 µM (CQ), and co-treated with CQ (4 µM + 25 µM) stained with PI after 24 h of nutrient deprivation. **e** Fluorescence analysis of the AO assay in nutrient-deprived PC-3 cells. **f** Fluorescence images of the AO assay in nutrient-deprived PC-3 cells. Cells were exposed to SSC185 (4 µM), CQ (25 µM), or the combination SSC185 (4 µM) + CQ (25 µM). Two control groups were included in this experiment: a control group under standard culture conditions and another group under nutrient-deprived conditions. Data were compared by analysis of variance (ANOVA) followed by Tukey’s post-hoc test. The data are representative of three independent experiments. Bars, S.E.M.; *p < 0.05, **p < 0.01, ***p < 0.001. AO: acridine Orange; CQ: chloroquine; ND: Nutrient deprivation; PI: propidium iodide

Cell-cycle distribution and plasma membrane integrity were evaluated in PC-3 cells under nutrient-deprived conditions following treatment with SSC185. Plasma membrane integrity was assessed based on the proportion of PI− cells, which correspond to cells retaining the ability to exclude propidium iodide. The PI− population was 93.40% ± 0.39 in FBS-supplemented controls and 85.12% ± 1.99 in nutrient-deprived controls. Treatment with 4 µM SSC185 under nutrient deprivation significantly reduced the proportion of PI− cells to 63.70% ± 4.39 (*p* < 0.001), indicating a reduction in the population of cells with preserved plasma membrane integrity (Fig. 8a, c). CQ (25 µM) similarly reduced the PI− population to 69.80% ± 3.26 (*p* ≤ 0.01), while co-treatment with SSC185 and CQ further reduced this population to 54.25% ± 2.13 (*p* < 0.01) compared with CQ alone.

Analysis of cell-cycle distribution showed that treatment with 4 µM SSC185 did not significantly alter the proportion of cells in G_0_/G_1_, S, or G_2_/M phases compared with the corresponding controls under either FBS-supplemented or nutrient-deprived conditions. In contrast, CQ treatment under nutrient deprivation increased the proportion of cells in G_0_/G_1_ (46.93% ± 2.38; *p* ≤ 0.05) and reduced the S-phase fraction (16.48% ± 2.33; *p* ≤ 0.05), indicating an altered cell-cycle distribution under this experimental condition.

### SSC185 does not increase acidic vesicle accumulation under nutrient deprivation

To evaluate changes in acidic vesicular compartments under nutrient-deprived conditions, PC-3 cells were analyzed by acridine orange staining following treatment with SSC185. The fluorescence pattern was similar to that observed under standard culture conditions, as SSC185 did not significantly increase the mean fluorescence intensity in the RFP channel (Fig. 8e, f). In contrast, CQ treatment resulted in a significant increase in RFP fluorescence (*p* < 0.05), consistent with greater accumulation of acridine orange-stained acidic vesicular compartments. Co-treatment with SSC185 and CQ produced a non-significant increase in RFP fluorescence compared with the corresponding control condition. These findings indicate that SSC185 does not promote detectable accumulation of acidic vesicular compartments under nutrient deprivation.

### SSC185 exhibits time-dependent thiol reactivity

**Fig. 9:**
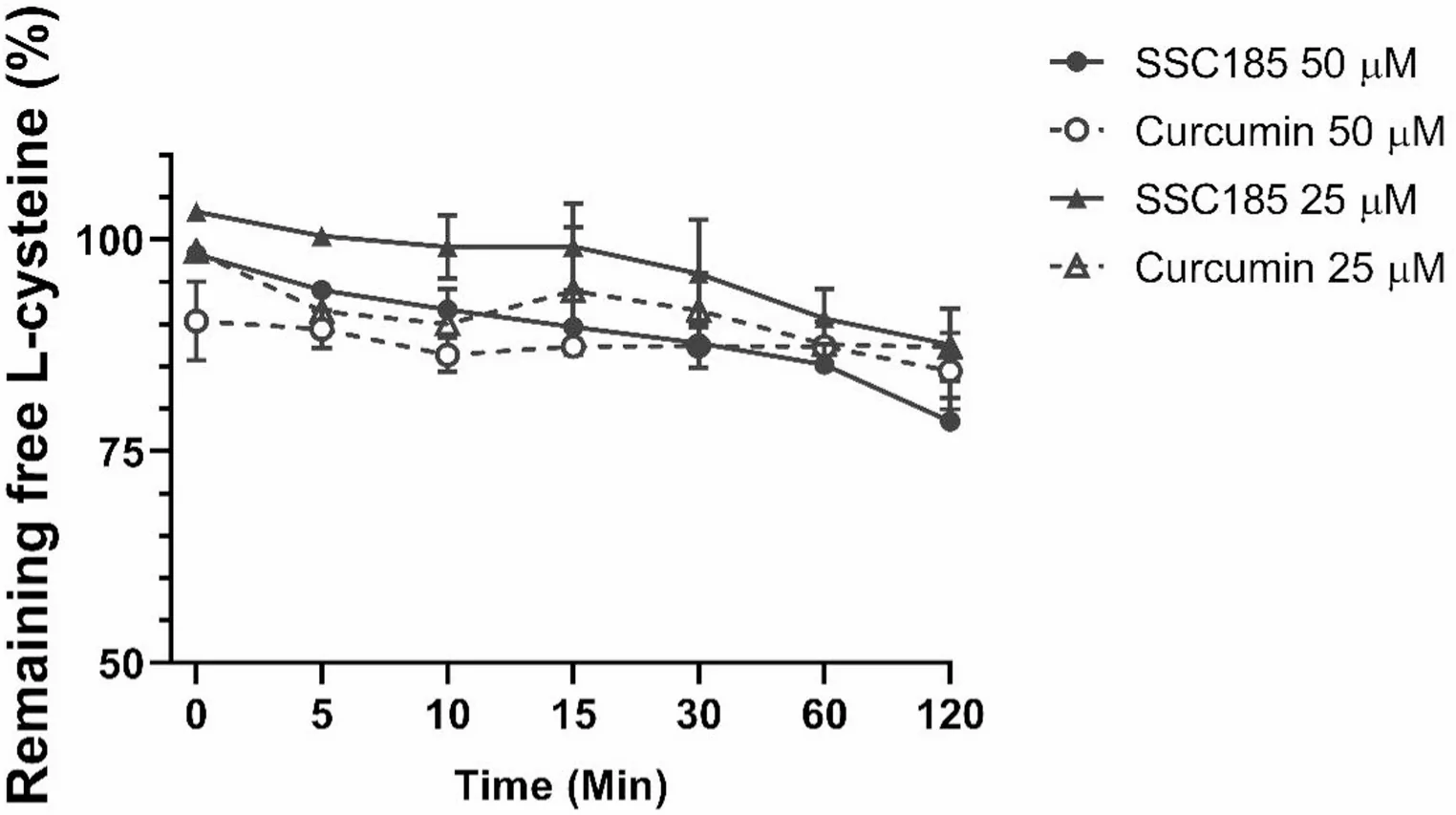
SSC185 exhibits moderate time-dependent reactivity to thiols. Reactivity of SSC185 to thiols as a function of time, evaluated using a DTNB-based L-cysteine assay. SSC185 or curcumin (25 µM or 50 µM) was incubated with L-cysteine (250 µM) in phosphate buffer pH 7.4 for the indicated time intervals, and the remaining levels of free L-cysteine were quantified using the DTNB/Ellman reagent at 405 nm. Data are expressed as a percentage of remaining free L-cysteine relative to the corresponding L-cysteine control, after correction for background and compound absorbance. Curcumin was used as an electrophilic reference compound. The data are representative of experiments in triplicate. Bars, S.E.M

To further assess the possibility of nonspecific electrophilic reactivity, a DTNB-based thiol-reactivity assay was performed with L-cysteine under physiologically relevant conditions. SSC185 induced a mild-to-moderate and time-dependent reduction in free L-cysteine levels, with 78.50% ± 2.78 of thiol content remaining after 120 min at 50 µM. A weaker effect was observed at 25 µM (87.55% ± 4.25), suggesting partial concentration dependence. Importantly, the overall profile was comparable to that observed for curcumin, the reference electrophilic compound (84.46% ± 4.53 and 87.28% ± 0.66 at concentrations of 50 µM and 25 µM, respectively). These findings indicate that SSC185 exhibits measurable but not rapid or exhaustive thiol reactivity under the tested conditions (Fig. 9).

## Discussion

In this study, we investigated the effects of the sulfonamide-chalcone hybrid SSC185 on androgen-independent PC-3 prostate cancer cells and observed that its cellular effects depend on exposure time, concentration, and nutritional conditions. The greater response observed under nutrient deprivation highlights the influence of the nutritional context on SSC185 activity and suggests that cellular adaptation to limited nutrient availability may shape the response of PC-3 cells to the compound [42, 43].

From a structural standpoint, the sulfonamide moiety exhibits distinctive electronic properties, acting simultaneously as a hydrogen-bond donor and acceptor, which favors stable interactions with diverse biomolecular targets [44]. In addition, the presence of two highly electronegative oxygen atoms bound to sulfur confers pronounced polarity to this fragment, a feature commonly associated with protein and enzyme recognition [45, 46]. The chalcone scaffold, in turn, contains an α,β‑unsaturated carbonyl group recognized as a prototypical Michael acceptor [47, 48]. This electrophilic center can form covalent adducts with cellular nucleophiles, particularly cysteine residues in regulatory proteins and metabolic enzymes. Consistent with this structural feature, SSC185 displayed measurable and time-dependent thiol reactivity in a DTNB-based assay using L-cysteine under physiological-like conditions, indicating the ability to interact with sulfhydryl-containing molecules without inducing rapid or exhaustive thiol depletion [49, 50]. Such covalent modifications may alter protein function, stability, or recognition by protein quality control systems, thereby disrupting cellular homeostasis [51, 52]. While electrophilic chalcone-containing compounds may potentially interact with multiple nucleophilic targets, selectivity in these systems is often influenced by binding orientation, target-site microenvironment, and the balance between reversible and irreversible interactions [53]. Therefore, additional studies will be important to further characterize target engagement and molecular selectivity associated with SSC185. In tumor cells, the formation of these adducts has been associated with dysregulation of redox‑sensitive signaling pathways, impairment of energy metabolism, and accumulation of misfolded or damaged proteins, contributing to proteotoxic stress and activation of cell death mechanisms [52, 54, 55]. Although α,β-unsaturated systems are sometimes associated with pan-assay interference compounds (PAINS) due to their potential electrophilic reactivity, increasing evidence indicates that not all Michael acceptors behave as nonspecific promiscuous agents, and several clinically relevant compounds contain related electrophilic motifs [56, 57]. In this context, the moderate and progressive thiol reactivity profile observed for SSC185, combined with its biological effects, contradicts the hypothesis of a mechanism driven solely by indiscriminate thiol sequestration. Thus, the hybrid structure of SSC185, combining a sulfonamide motif commonly linked to enzyme modulation with a conjugated aromatic system bearing Michael acceptor properties and electronegative substituents that tune electronic and lipophilic profiles, suggests the potential for multitarget cellular interactions [58–60].

Our comparison of continuous and intermittent exposure protocols showed that short treatments were insufficient to elicit robust cytotoxicity, whereas prolonged exposures resulted in a greater reduction in MTT signal and clonogenic capacity. The loss of proliferative potential observed in clonogenic assays, particularly after 24 h of treatment, even at lower concentrations, suggests that SSC185 induces irreversible cellular damage, likely associated with loss of metabolic competence and eventual activation of late cell death programs [61, 62]. These findings support the notion that SSC185 exerts a time‑dependent, cumulative effect, consistent with intracellular accumulation and progressive stress induction rather than an immediate cytotoxic insult [63, 64]. Similar behavior has been reported for sulfonamides and chalcone derivatives acting as modulators of cellular homeostasis [65, 66], survival pathways [67–71], energy metabolism [72–75], and mitochondrial integrity [76–78].

SQSTM1/p62 is a multifunctional adaptor protein that links LC3B to ubiquitinated substrates, thereby promoting their sequestration into autophagosomes for degradation [79, 80]. In many contexts, simultaneous LC3B-I-to-LC3B-II conversion and p62 accumulation indicate an aborted or inefficient autophagic response, often reflecting a late blockade of autophagic flux [9, 81–83]. In the present study, SSC185 induced a significant increase in SQSTM1/p62 and LC3B-I levels after 24 h of treatment, without a detectable change in LC3B-II or a significant increase in acridine orange-stained acidic vesicles. This pattern indicates that SSC185 alters the expression profile of autophagy-related markers; however, it does not allow definitive conclusions regarding autophagic flux. In particular, SQSTM1/p62 accumulation may result from different cellular processes, including changes in its synthesis, degradation, or stress-responsive regulation, and therefore cannot be interpreted solely as evidence of impaired autophagic degradation [84, 85]. Similarly, the absence of detectable LC3B-II accumulation does not exclude alterations in autophagic activity, since LC3B-II levels reflect the balance between its formation and degradation. Thus, the molecular changes observed following SSC185 treatment may reflect a cellular stress-associated response involving autophagy-related proteins, although their functional relationship with autophagic flux remains to be established. Dynamic approaches, including time-course analyses and direct measurements of autophagic flux, would be required to determine the mechanistic significance of these alterations.

In this context, it is important to consider that SQSTM1/p62 is a multifunctional adapter protein involved in various cellular stress response pathways through its multiple protein-interaction domains [26, 86]. Previous studies have demonstrated that p62 expression can be modulated in response to oxidative and proteotoxic stress and is functionally linked to stress-responsive pathways, including the KEAP1/NRF2 axis [26, 87–90]. Against this backdrop, the increase in SQSTM1/p62 levels observed following SSC185 treatment may represent a stress-associated cellular response. The morphological changes observed after exposure to SSC185, including cytoplasmic vacuolization and loss of plasma membrane integrity, corroborate the occurrence of cellular injury but do not establish a direct link to p62 accumulation [91–93]. Thus, current data do not determine whether p62 accumulation contributes functionally to SSC185-induced cytotoxicity or represents a secondary response to cellular stress. Furthermore, inhibition of protein synthesis with cycloheximide did not substantially alter the response to SSC185 under the conditions evaluated, suggesting that ongoing protein synthesis is not required for the observed effect [94, 95]. Consequently, these findings indicate that SSC185 treatment is associated with alterations in markers related to stress and proteostasis; however, the functional contribution of SQSTM1/p62 and the specific mechanisms underlying these changes remain to be determined.

Data from the literature highlight the importance of the PI3K/AKT/mTOR pathway in many cellular processes, contributing especially to the growth and survival of various tumors [96–98], and this pathway is frequently hyperactivated in prostate cancer [99–102]; Notably, 4E-BP1 is frequently hyperphosphorylated in CRPC, allowing for high cap-dependent translational activity via eIF4E [103, 104]. In the exploratory immunoblot analysis, SSC185 treatment was associated with reduced AKT and total 4E-BP1 levels, whereas phosphorylated 4E-BP1 remained relatively unchanged. Although these observations may indicate differential regulation of signaling pathways involved in cell growth and protein synthesis, their exploratory nature precludes quantitative interpretation. In contrast, the reduction in p-RPS6 was consistently observed in subsequent independent experiments, supporting an effect of SSC185 on this downstream signaling component. Likewise, the increase in p-ERK1 may be consistent with the activation of a compensatory stress-response mechanism [105], although its functional significance remains to be established. These molecular alterations were accompanied by time-dependent changes in cell-cycle distribution and a progressive reduction in clonogenic capacity. At earlier exposure times, alterations in cell-cycle distribution were observed without comparable loss of long-term proliferative capacity at lower SSC185 concentrations, whereas prolonged exposure resulted in a more pronounced and persistent reduction in clonogenic potential. Previous studies have shown that inhibition of the PI3K/AKT axis can reduce RPS6 phosphorylation and alter cell-cycle progression and the expression of cell-cycle regulatory proteins in prostate cancer cells, supporting an important role for PI3K/AKT/mTOR signaling in the maintenance of prostate cancer cell proliferation [106–108]. Nevertheless, although the signaling alterations observed here occurred alongside the progressive cellular effects of SSC185, the present data do not establish a direct causal relationship between these molecular changes and the development of sustained cytotoxic effects.

Evaluation of SSC185 under standard and nutrient-deprived conditions revealed a marked influence of the nutritional context on the cellular response to the compound. Under standard culture conditions, pharmacological modulation of autophagy, protein synthesis, apoptosis, or redox status did not substantially alter the response to SSC185. In contrast, nutrient deprivation markedly potentiated SSC185 activity, reducing the concentration required to affect the MTT signal. Under this condition, pre-treatment with NAC partially attenuated the effect of SSC185. Although NAC is widely used as an antioxidant and a precursor for intracellular cysteine and glutathione, its protective effects may also involve direct interactions with reactive electrophilic species [109, 110]. Therefore, the partial protection conferred by NAC cannot be interpreted as direct evidence that oxidative stress mediates SSC185 activity. Mitochondrial membrane potential also decreased during prolonged exposure and under nutrient-deprivation conditions, indicating that mitochondrial dysfunction accompanies the cellular response to SSC185. Although these findings demonstrate that nutrient deprivation increases the sensitivity of PC-3 cells to SSC185 and that this intensified response is associated with alterations in redox-sensitive and mitochondrial parameters, further studies are needed to directly assess cellular metabolism and determine the metabolic processes involved.

## Conclusion

Taken together, the results of this study demonstrate that SSC185 exerts time- and concentration-dependent effects on androgen-independent PC-3 prostate cancer cells. These effects were accompanied by alterations in cell cycle distribution, reduced AKT/RPS6 signaling, and increased levels of SQSTM1/p62 and LC3B-I without detectable accumulation of LC3B-II or increased formation of acidic vesicles, as well as a delayed compromise of mitochondrial membrane potential. Notably, nutrient deprivation markedly potentiated the cellular response to SSC185, lowering the concentrations required to affect the MTT signal and clonogenic potential. In summary, the data suggest that SSC185 activity is influenced by the cellular nutritional context and involves coordinated changes in signaling, stress-related proteins, and mitochondrial function. Further investigation into these interconnected cellular responses may help elucidate the molecular processes underlying the enhanced effects of SSC185 under nutrient-deprived conditions.

Although additional studies will be necessary to further characterize target selectivity, pharmacological safety, and the molecular mechanisms underlying SSC185 activity, including orthogonal approaches to evaluate covalent interactions and protein engagement, the present findings support the potential of sulfonamide-chalcone hybrids as promising pharmacological scaffolds capable of compromising adaptive stress responses and proliferative capacity in aggressive prostate cancer cell models.

## Data Availability

No datasets were generated or analysed during the current study.
